# A systematic review of exercise intervention reporting quality and dose in studies of intermittent claudication

**DOI:** 10.1177/17085381211070700

**Published:** 2022-02-07

**Authors:** Saïd Ibeggazene, Sean Pymer, Stefan T Birkett, Edward Caldow, Amy E Harwood

**Affiliations:** 1 College of Health, Wellbeing and Life Sciences, 7314 Sheffield Hallam University, Sheffield, UK; 2 Academic Vascular Surgical Unit, 12195 Hull York Medical School, Hull, UK; 3 School of Sport and Health Sciences, 6723 University of Central Lancashire, Preston, UK; 4 School of Health and Society, 7046 University of Salford, Salford, UK; 5 Centre for Sports, Exercise and Life Sciences, 120958 Coventry University, Coventry, UK

**Keywords:** Intermittent claudication, systematic review, exercise, exercise therapy

## Abstract

**Background:**

Exercise therapy is an important treatment option for people with intermittent claudication (IC). Appropriate reporting of exercise interventions in populations with IC within randomised controlled trials (RCTs) is important to ensure that research can be translated into clinical practice. Therefore, the purpose of our review is to evaluate the reporting of exercise interventions in RCTs of exercise therapy in patients with IC.

**Methods:**

A systematic search was performed to identify relevant trials in patients with IC published until May 2020. Studies including only participants with critical limb ischaemia or asymptomatic peripheral artery disease were excluded. Each trial was scored using the recently developed ‘Consensus on Exercise Reporting Template’ (CERT) which has a maximum obtainable score of 19.

**Results:**

Of 1489 unique records identified from the search, 73 trials were included, reporting 107 exercise interventions. Overall, the average CERT score was 10/19. The exercise equipment used, the use of supervision and a description of whether the exercise prescription was tailored or generic were the most frequently reported intervention components. The motivational strategies used, intervention adherence and intervention fidelity were the most underreported CERT components. There was no trend indicating that CERT scores were higher in more recent publications.

**Conclusions:**

We have identified that important details about exercise interventions are frequently missing from the published literature. These missing data hinder replication of research findings and limit the translation of evidence into clinical practice.

## Introduction

Peripheral arterial disease (PAD) is characterised by atherosclerosis of the arteries supplying the lower limbs, resulting in a reduced blood supply. The prevalence of PAD is estimated to have increased by 23.5% between 2000 and 2010^1^ with current estimates at 237 million people affected globally.^2^ Around 20–25% of individuals over 60 years old experience symptoms from PAD,^3^ most commonly intermittent claudication (IC). IC is a reproducible leg pain or discomfort that manifests during physical exertion, typically walking, and is relieved by rest. IC has deleterious effects on quality of life and is associated with an increased mortality risk.^4^

A first-line treatment for IC is exercise therapy, a prescription of regular supervised exercise to improve quality of life via improvements in walking performance. The efficacy of exercise therapy for improving walking performance is supported by Level 1A evidence.^5^ As such, supervised exercise training is recommended for the management of IC by the European Society for Vascular Surgery and European Society of Cardiology,^6^ the UK National Institute for Health and Care Excellence^7^ and the American Heart Association.^8^

Notwithstanding evidence supporting the efficacy of exercise therapy in clinical trials, its effectiveness (i.e. real-world treatment effect) is less clear. Shortcomings with service provision,^9,10^ programme uptake and adherence^11^ are amongst the known factors that have limited the effectiveness of simply recommending exercise therapy. Despite this, how it is implemented in practice is poorly understood, precluding the advancement of practical guidance. Current guidance is based on highly heterogenous literature in terms of the treatment context, healthcare professional training/discipline, population characteristics and exercise prescription - the frequency, intensity, time and type of exercise which together constitute the dose of exercise received.^12^ Whilst it is encouraging that in a pooled analysis, exercise has a meaningful benefit in this population, there remains a challenge for distilling knowledge about how to optimally provide this key treatment.

Very few published studies have evaluated the effectiveness of exercise programmes in routine care. Underlying this is the reality that exercise therapy is a complex intervention; multiple components within an exercise prescription interact to achieve an effective exercise dose which must be sustained for an adequate period to achieve the desired therapeutic effect. Exercise interventions therefore require detailed descriptions to enable efficacious research protocols to be faithfully implemented in practice and to inform robust evaluations of exercise services.

To understand and reliably reproduce the effects of an exercise protocol used in a trial, sufficient detail regarding how the intervention was conducted must be provided. Therefore, this review aimed to evaluate the quality of reporting of published exercise interventions used to treat IC in randomised controlled trials (RCTs). Collating the components of published exercise interventions also will allow us to clarify the inferences that can be made from available data about exercise programming and prescription for people with IC. This will enable us to identify future research priorities in this field.

## Methods

This review was conducted in line with the Preferred Reporting Items for Systematic Review and Meta-Analysis (PRISMA) guidance.^13^

### Search strategy

Four databases, CINAHL, Medline, EMBASE and Cochrane CENTRAL, were searched from 1995 to May 2020. In addition, five existing systematic reviews and meta-analyses were manually searched to identify other trials eligible for inclusion.^5,14–17^ Only studies published in the English language and relating to adults with IC (over 18 years of age) were included. Titles and abstracts were independently interrogated for inclusion by two reviewers (SB and SP) and disagreements resolved by discussion. The full text of any potentially eligible article was then screened against the inclusion and exclusion criteria. Full search strategies can be found in Supplementary material 1.

### Eligible articles

We included prospective RCTs where patients with IC (typical and atypical) were randomised to at least one arm that included a structured supervised or unsupervised exercise programme. We defined a structured exercise training programme as one that stated the prescribed frequency, intensity and/or duration. No limits were placed on the type or duration of the exercise intervention. We elected to exclude studies that were published prior to 1995 as the majority of exercise programmes published after this date were designed using the recommendation of a specific meta-analysis.^18^ Studies including patients with critical limb ischaemia or asymptomatic PAD were also excluded.

### Outcomes

To assess the quality of the reporting of the exercise intervention used in these trials, the ‘Consensus on Exercise Reporting template’ (CERT) was used.^19^ The CERT was developed and endorsed by an international panel of experts to allow a standardised appraisal of published exercise rehabilitation interventions. It comprises a 16-item checklist that was designed to evaluate the completeness of reporting of exercise descriptions and spans the ‘who’, ‘what’, ‘when’, ‘where’ and ‘how’ of exercise interventions. We utilised the CERT ‘Explanation and Elaboration Statement’ to inform scoring.^19^ Each item of the CERT was scored as a binary outcome (adequately reported vs inadequately reported, unclear or not reported at all) with a maximum possible score of 19.

### Data extraction

Five assessors (SI, SB, EC, SP and AH) independently reviewed and extracted data using a standardised, purpose-built database. Where a study included more than one intervention arm which involved exercise, data were extracted for each arm and the individual intervention was evaluated rather than only the study. Extraction for each study was cross checked for accuracy and completeness by two reviewers (SI and AH). Data extraction included study characteristics, sample size, description of exercise prescription according to the ‘FITT principle’ (frequency, intensity, time and type of exercise performed) and information related to each CERT item.^19^ In addition, whilst the first CERT item considers whether a description of the exercise equipment is provided, we also recorded whether the make or model of equipment was reported, but this did not contribute to the overall CERT score. Where applicable, we consulted additional study sources (i.e. protocols and supplementary materials) to aid scoring.

### Data synthesis

A narrative synthesis of the reporting of exercise interventions was performed. Intervention content was summarised by item according to the CERT checklist and FITT descriptors. To examine the change in intervention reporting quality over time, a Spearman correlation coefficient was calculated between the year of study publication and a study’s CERT score. Alpha was accepted as *p* < 0.05.

## Results

**Figure 1.**
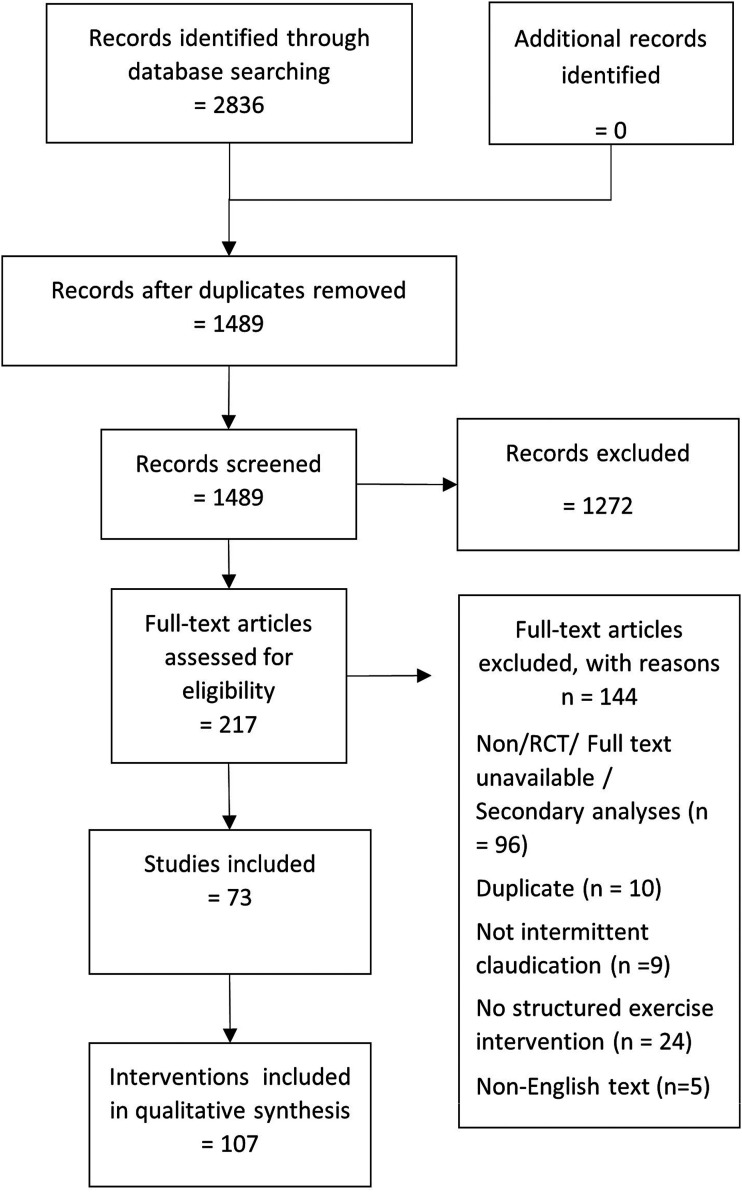
PRISMA flow diagram.

Database searches identified 1489 unique records. Of these, 73 trials, comprising 107 exercise interventions, met the^20^ inclusion criteria and were ultimately included in this review (Figure 1).^21–87^

### Consensus on exercise reporting template score

**Figure 2.**
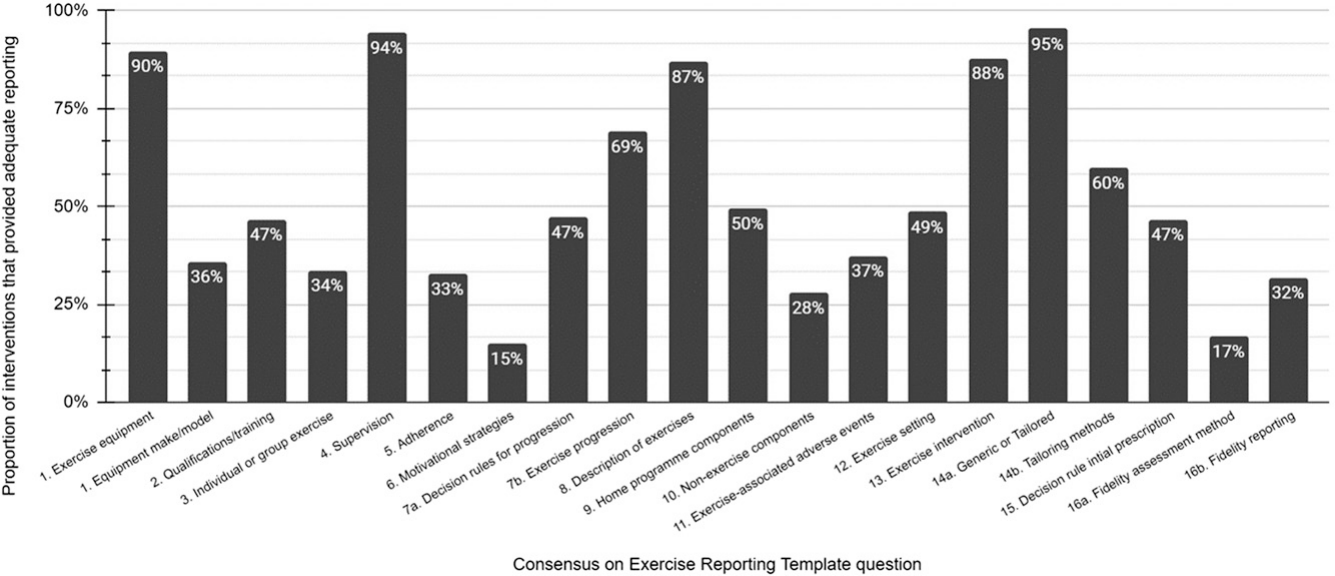
Reporting standards by individuals question of the Consensus on Exercise Reporting Template (CERT) in exercise interventions to treat intermittent claudication.

**Figure 3.**
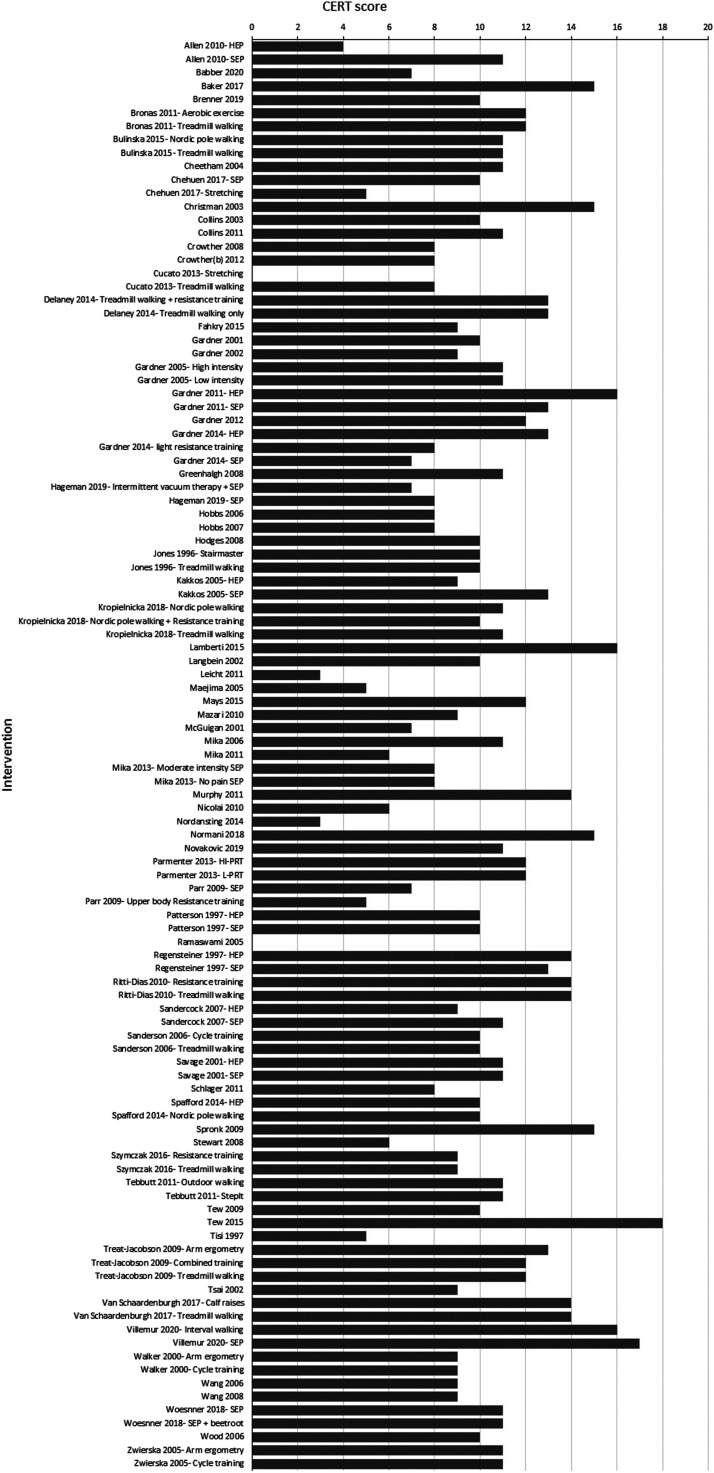
Reporting standards by intervention for the Consensus on Exercise Reporting Template (CERT) in exercise interventions to treat intermittent claudication. For studies that have multiple exercise intervention arms the intervention being scored has been disambiguated.

A summary of the scores for each CERT item is provided in Figure 2. The mean CERT score was 10 ± 3 out of a possible 19. The CERT score for each intervention is displayed in Figure 3. Only 28% of interventions scored more than 11/19. There was no difference between the CERT scores in the 11 studies published after the CERT guidance was released and those that pre-dated the CERT (11.3 ± 3.3 vs 9.9 ± 3.2; *p* = 0.127). There was no relationship between year of publication and CERT score (*ρ =* 0.14, *p* = 0.14, Supplemental Figure 1).

Question 1: Detailed description of the type of exercise equipment.

The mode of exercise performed was typically described with an indication of the type of equipment used (if any) such as a treadmill or Nordic walking poles. However, only 36% of studies that described the use of equipment gave specific details of the make or model used.

Question 2: Detailed description of the qualifications, expertise and/or training.

Less than half (47%) of the included interventions provided a description of the qualifications, profession and/or training of those delivering the exercise intervention. A variety of professions were described including physiotherapists (most common), vascular nurses, research nurses, exercise physiologists, exercise instructors, rehabilitation assistants, vascular technologists and research assistants.

Question 3: Describe whether exercises are performed individually or in a group.

Information regarding whether interventions were delivered in a group or individually was limited, with only 34% of interventions providing this specific information.

Question 4: Describe whether exercises are supervised or unsupervised; how they are delivered.

The vast majority (94%) of interventions reported the level of supervision provided in each intervention.

Question 5: Detailed description of how adherence to exercise is measured and reported.

Few interventions (33%) provided a description of how they defined adherence to the intervention. If adherence was measured, it was typically via self-reported activity logs or records of attendance to supervised sessions.

Question 6: Detailed description of motivation strategies.

Very few interventions (15%) described the use of behavioural or motivational strategies to support adherence to the intervention. Examples include providing information about the benefits of exercise via written materials or having weekly telephone contact with a nurse or exercise professional who provided support adhere to the intervention.

Question 7a: Detailed description of the decision rule(s) for determining exercise progression; Question 7b: Detailed description of how the exercise programme was progressed.

A decision rule determining how the dose of exercise would be progressed based on an individuals’ performance was provided in less than half of the interventions (47%); for example, increasing the speed or elevation of a treadmill when a participant walked for 8 min without reaching moderate pain. Occasionally, a general rule for exercise dose progression was employed irrespective of individual performance, such as increasing the duration of walking in a session by 5 min every 2 weeks. Accordingly, descriptions of how exercise was progressed were better reported (69%). Progression was typically made by increasing the exercise intensity (e.g. the speed or gradient of treadmill walking) or total duration.

Question 8: Detailed description of each exercise to enable replication.

An adequate description of the exercises that made up the intervention that would enable replication was provided in most instances (87%). However, it was noted that many instructions were imprecise and could be interpreted and implemented in various ways. For example, where multiple exercises were used within an intervention, it was often unclear how vigorous an effort one should make for different exercises, whether the order of exercises was fixed or variable, or whether rest periods were used within or between exercise bouts.

Question 9: Detailed description of any home programme component.

Half of the interventions described a home-based component such as completing the entire programme at home or supplementing centre-based activities with unsupervised walking in another setting of the participant’s choosing.

Question 10: Describe whether there are any non-exercise components.

Additional intervention components such as the provision of written or verbal advice regarding diet, weight loss, physical activity or smoking cessation were infrequently reported (28%). Other examples include specification of the standard of medical care in study participants such as the provision of antiplatelet and lipid-lowering therapies.

Question 11: Describe the type and number of adverse events that occurred during exercise.

Reporting of adverse events was low (37%). Most studies that commented on adverse events stated that none occurred. In some instances, only unanticipated or serious adverse events were reported. Of the studies that reported adverse events, most did not specify whether an adverse event was related to the intervention - only one related event was reported (musculoskeletal injury). Other instances of adverse events were not adequately described to identify whether they were caused by the intervention.

Question 12: Describe the setting in which the exercises are performed.

Less than half of the interventions (47%) described the environment (gym, laboratory, outdoors, etc.) where exercise was performed.

Questions 13: Detailed description of the exercise intervention; 14a: Describe whether the exercises are generic (one-size-fits-all) or tailored; 14b: Detailed description of how exercises are tailored to the individual.

Most studies provided a detailed description of the exercise intervention (88%) and provided information as to whether the exercise prescription was generic or individually tailored (95%). Only 60% of interventions provided a detailed description of how exercise was individually tailored.

Question 15: Describe the decision rule for determining the starting level.

Only 47% of interventions described a decision rule that was used to determine the initial exercise dose prescribed to a participant, such as walking at 75% of the workload achieved on a treadmill test.

Question 16a: Describe how adherence or fidelity is assessed/measured; 16b: Describe the extent to which the intervention was delivered as planned.

It was rarely reported (17%) that adherence to the intervention was assessed. In most instances, where fidelity was considered, the limited definition of attendance to training sessions was used. This assumes that the delivery of exercise during sessions is perfect or very consistent. Only 32% of interventions reported that they were delivered as planned - either in writing or via reported data.

### Frequency, intensity, time and type of exercise performed descriptors

#### Frequency

The most common exercise frequency was 3 times per week (49/107); followed by daily exercise (29/107); two sessions per week (23/107); one session per week (3/107); and four, five or six sessions per week (1/107). Fourteen interventions used complex prescriptions such as 2–3 sessions per week, at least 3 sessions per week, 3 sessions per day or had a variable frequency over the course of the programme.

#### Intensity

Ten different categorisations of prescribing exercise intensity were employed. The most common prescription was a description of claudication pain intensity (28/107). The intensities ranged from the onset of claudication pain to maximal pain. However, the terms used to describe these intensities varied considerably. Terms such as ‘near pain threshold’, ‘till claudication was noted’, ‘moderately severe pain’, ‘submaximal pain’, ‘intense pain’, ‘near max pain’ and ‘unbearable pain’ were used. Two different scales were used to prescribe exercise by the intensity of claudication pain experienced: the ACSM scale which ranges from 1 to 4 (11/107) and the claudication pain scale (14/107) which ranges from 1 to 5. Treadmill tests were used to individually prescribe a treadmill-specific workload in 16/107 interventions. Six interventions prescribed ergometer workloads (cycle or arm) from an ergometer test. Ten interventions prescribed intensity based on rating of perceived exertion and two based on an individual’s heart rate. Seven interventions prescribed resistance exercise via fixed loads, fixed repetition numbers, isokinetic dynamometer-based loads or as a percentage of one repetition maximum. Intensity prescriptions were not clearly specified in 11 interventions with some based on walking a ‘maximum distance’ or walking ‘to tolerance’ if reporting an intensity prescription at all.

#### Time

The duration of exercise sessions was reported in almost all interventions (102/107) with some interventions prescribing completion of a volume of exercise without an indication of duration. Most interventions prescribed were for a duration of 30–59 min (63/107) or ≥ 60 min (21/107).

#### Type

Treadmill walking was the most common modality of exercise prescribed (53/107), followed by outdoor/overground walking (19/107), circuit training (8/107), resistance training (6/107), Nordic pole walking (6/107), cycling (5/107) or arm ergometry (3/107). Three interventions did not report the type of exercise prescribed.

## Discussion

The aim of this study was to evaluate the quality and completeness of reporting in published exercise interventions for patients with IC which has been defined by the CERT^19^ and exercise dose according to the FITT principles. Overall, we identified 107 exercise interventions from 73 studies that adopted a variety of exercise modalities. Our main finding was that in general, the quality of reporting of exercise interventions for patients with IC was poor.

Only 8 out of 19 of the CERT criteria were reported in most interventions. The components that were well reported included the type of equipment used, supervision, a description of the exercises provided, a description of the exercise intervention and whether it was a tailored or generic programme. The highest CERT score was 18/19 which was attained by one intervention which only omitted to describe how fidelity was assessed.^88^ Furthermore, there was no trend to suggest intervention reporting quality had improved over the last 25 years.

The least reported aspect was the use of motivational strategies (question 6), described in only 15% of interventions. Whether this result is artificially low due to a reporting bias is unclear. Investigators may not realise the importance of reporting such strategies, even when they are used. Engaging patients with IC in exercise interventions is challenging, and poor uptake and adherence rates have been noted,^11^ often because patients desire a ‘quick-fix’ for their symptoms.^89^ Plausibly, the application of known facilitators to exercise behaviour such as goal setting, accessing support systems^90^ or many other potentially effective behaviour change techniques^91^ may improve adherence to exercise interventions. That the use of behavioural support strategies is seldom reported limits our understanding of how to promote adherence to exercise in clinical trials and routine care. This problem is compounded by poor reporting of exercise adherence.

The fidelity of, or adherence to, an exercise intervention was reported for only 17% of interventions (question 16a). These results are congruent with those of others using different reporting frameworks.^92^ Intervention fidelity is integral for determining the internal and external validity of intervention-based trials,^93,94^ is endorsed by the CONSORT recommendations^95^ and should be reported in all RCTs. This presents a significant potential confounder of pooled analyses of exercise interventions. Different treatment effects might be expected for interventions with 40% versus 80% adherence, but at present, we are largely unable to characterise this effect. Where intervention adherence was described, it was predominantly limited to a description of attendance to exercise sessions. More comprehensive reporting of adherence to an intervention should include a description of the exercise intensity achieved during training and total duration of exercise performed at the prescribed intensity.

Merely recording attendance is not a sufficient measure of intervention fidelity as this assumes that the exercise being performed is of an adequate intensity, type and duration (i.e. dosage) to elicit a benefit. Inadequate measurement and reporting of these components risks efficacious interventions being depicted as ineffective solely because of poor implementation. This could limit the support for this beneficial treatment and contribute to research waste. Such reporting issues have been identified for exercise interventions in hypertension,^96^ breast cancer^97^ and cardiac rehabilitation.^98^ Though exercise intensity was frequently reported (89% of interventions), ten different methods of prescription were used. There is limited consensus on what exercise intensity should be prescribed, with professional societies recommending walking to mild to moderate,^99^ moderate,^3^ near-maximal^100^ and maximal pain.^7^ This heterogeneity, along with poor reporting of adherence, poses a major challenge for between-study comparison of exercise interventions. There is a clear need for standardisation of the prescription and reporting of exercise intensity in this population.

The other principles of exercise prescription include frequency, time and type.^101^ Clear reporting of these components is vital to allow replication of interventions and translate research knowledge into clinical guidelines for exercise. Of the 107 interventions included in this review, all of them adequately reported the frequency of exercise. Forty-nine prescribed a frequency of three times per week, in line with most current guidance.^12^ Time was reported in 102/107 interventions (95%) and was predominantly 30–59 min in duration. Only 3/107 interventions did not describe the type of exercise prescribed. Our results suggest that these components are well reported in the IC literature, though they may not conform to the available clinical guidelines.

Finally, the inclusion of non-exercise components was another poorly reported aspect of exercise interventions. These components may include dietary advice, counselling or patient education with regard to medication adherence or smoking cessation. Only 28% of interventions provided any details of these components, which may plausibly influence treatment outcomes. Again, it is unclear whether these components are underreported or simply absent from most interventions.

Evidently, there is a need to improve reporting quality in this field; at present, there are few tools available to achieve this. The CONSORT checklist is one such tool that has improved the reporting of aspects of RCTs,^102^ though more detailed definitions of intervention adherence may be required for complex interventions. Of the 16 (22%) studies that referred to the CONSORT reporting guidance in their manuscripts, only 4 attempted to adhere to this guidance (beyond the inclusion of a CONSORT diagram). No study included in this review made reference to the use of the CERT checklist or the TiDieR checklist. A prudent recommendation would be to require study authors to submit a research checklist as a supplementary material to improve the quality of reporting of exercise interventions in IC populations. The CERT is a comprehensive tool that specifies many important aspects of exercise interventions that should be reported; however, at present, it lacks IC-specific criteria pertaining to the exercise prescription. As such, we recommend that a novel checklist should be developed and trialled to examine the effect of an IC-specific research checklist for exercise interventions.

### Limitations

By design, this review has only been able to describe the quality of reporting in the IC literature. It has not been able to investigate reasons for the observed shortcomings in reporting. Some details may be omitted due to word limits or a perceived lack of importance. Whether requiring greater detail in the reporting of exercise interventions in this population will increase the publication of trial protocols or cause the omission of other important information in manuscripts is unknown.

## Conclusion

The reporting of exercise interventions in populations with IC is poor. In particular, the reporting of adherence to interventions, strategies to motivate individuals to exercise and non-exercise components of the interventions were rarely reported. Additionally, many different descriptions of exercise intensity were used which will hinder between study comparison. As such, standardisation of the prescription and reporting of exercise intensity in studies including patients with IC is essential. A concerted effort is needed on the part of researchers, reviewers and journal editors to improve the quality of reporting of key aspects of exercise interventions to facilitate the advancement of methodological rigour in this area.

## Supplemental Material

sj-pdf-1-vas-10.1177_17085381211070700 – Supplemental Material for A systematic review of exercise intervention reporting quality and dose in studies of intermittent claudication Click here for additional data file. Supplemental Material, sj-pdf-1-vas-10.1177_17085381211070700 for Predictors A systematic review of exercise intervention reporting quality and dose in studies of intermittent claudication by Saïd Ibeggazene, Sean Pymer, Stefan T Birkett, Edward Caldow and Amy E Harwood in Vascular
