# Supplementary material for: A systematic review of exercise intervention reporting quality and dose in studies of intermittent claudication
Source: Vascular. 2022 Feb 7;31(3):477–88. doi: 10.1177/17085381211070700 (PMC10233510; doi:10.1177/17085381211070700)
Supplement: sj-pdf-1-vas-10.1177_17085381211070700 – Supplemental Material for A systematic review of exercise intervention reporting quality and dose in studies of intermittent claudication [file sj-pdf-1-vas-10.1177_17085381211070700.pdf]

Supplementary information:

Medline search terms

1. Exp. Peripheral vascular diseases
2. Exp. Peripheral arterial disease
3. Exp. Intermittent claudication
4. Maximum walking distance
5. Maximal walking distance
6. MWD
7. Maximal walking time
8. Maximum walking time
9. Exp. Exercise
10. Exp. Exercise therapy
11. Exp. Rehabilitation
12. Walking therapy
13. Treadmill
14. Training
15. Fitness
16. Plantar Flexion
17. Absolute Claudication Distance
18. ACD
19. MWT
20. Maximum claudication distance
21. Maximal claudication distance
22. Maximal claudication time
23. Maximum claudication time
24. MCT
25. MCD
26. Absolute claudication time
27. ACT
28. 1 OR 2 OR 3
29. 4 OR 5 OR 6 OR 7 OR 8 OR 17 OR 18 OR 19 OR 20 OR 21 OR 22 OR 23 OR 24 OR 25 OR 26 OR 27
30. 9 OR 10 OR 11 OR 12 OR 13 OR 14 OR 15 OR 16
31. 28 AND 29 AND 30

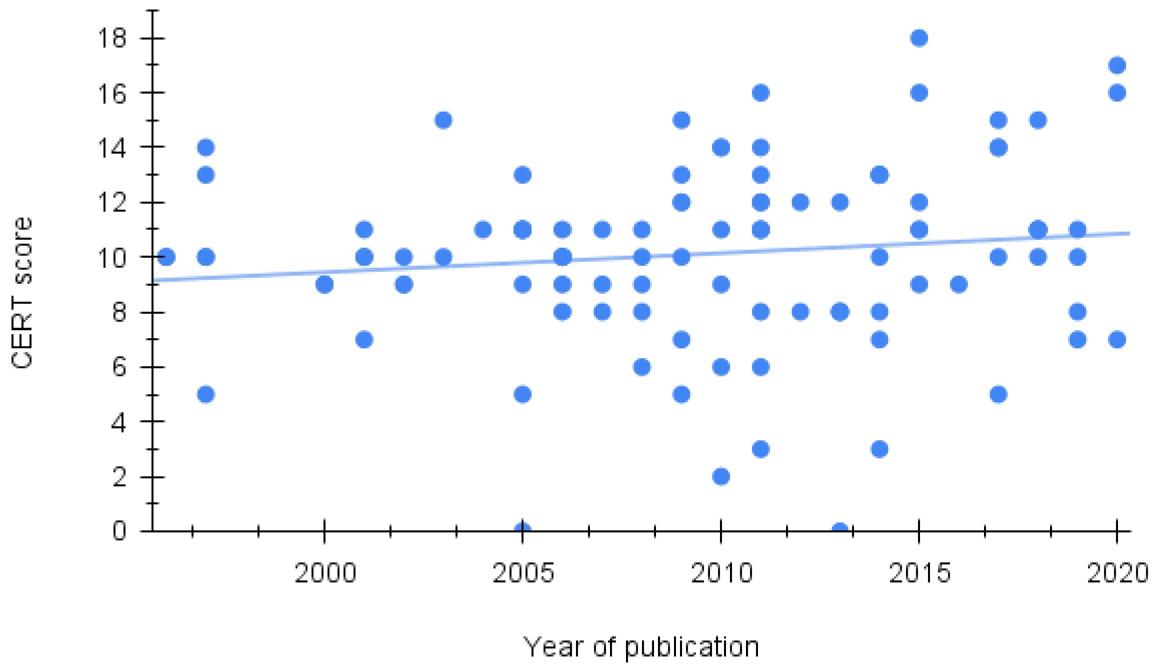

Section/topic	#	Checklist item
**TITLE**		
Title	1	Identify the report as a systematic review, meta-analysis, or both.
**ABSTRACT**		
Structured summary	2	Provide a structured summary including, as applicable: background; objectives; d criteria, participants, and interventions; study appraisal and synthesis methods; re implications of key findings; systematic review registration number.
**INTRODUCTION**		
Rationale	3	Describe the rationale for the review in the context of what is already known.
Objectives	4	Provide an explicit statement of questions being addressed with reference to part comparisons, outcomes, and study design (PICOS).
**METHODS**		
Protocol and registration	5	Indicate if a review protocol exists, if and where it can be accessed (e.g., Web ad registration information including registration number.
Eligibility criteria	6	Specify study characteristics (e.g., PICOS, length of follow-up) and report charac language, publication status) used as criteria for eligibility, giving rationale.
Information sources	7	Describe all information sources (e.g., databases with dates of coverage, contact additional studies) in the search and date last searched.
Search	8	Present full electronic search strategy for at least one database, including any lim repeated.
Study selection	9	State the process for selecting studies (i.e., screening, eligibility, included in syste included in the meta-analysis).
Data collection process	10	Describe method of data extraction from reports (e.g., piloted forms, independent processes for obtaining and confirming data from investigators.

Data items	11	List and define all variables for which data were sought (e.g., PICOS, funding sou [illegible] simplifications made.
Risk of bias in individual studies	12	Describe methods used for assessing risk of bias of individual studies (including s [illegible] done at the study or outcome level), and how this information is to be used in any [illegible]
Summary measures	13	State the principal summary measures (e.g., risk ratio, difference in means). [illegible]
Synthesis of results	14	Describe the methods of handling data and combining results of studies, if done, [illegible] (e.g., I^2) for each meta-analysis.

Section/topic	#	Checklist item
Risk of bias across studies	15	Specify any assessment of risk of bias that may affect the cumulative evidence (e [illegible] reporting within studies).
Additional analyses	16	Describe methods of additional analyses (e.g., sensitivity or subgroup analyses, [illegible] which were pre-specified.
**RESULTS**		
Study selection	17	Give numbers of studies screened, assessed for eligibility, and included in the re [illegible] each stage, ideally with a flow diagram.
Study characteristics	18	For each study, present characteristics for which data were extracted (e.g., study [illegible] provide the citations.
Risk of bias within studies	19	Present data on risk of bias of each study and, if available, any outcome level as [illegible]
Results of individual studies	20	For all outcomes considered (benefits or harms), present, for each study: (a) sim [illegible] intervention group (b) effect estimates and confidence intervals, ideally with a for [illegible]
Synthesis of results	21	Present results of each meta-analysis done, including confidence intervals and m [illegible]
Risk of bias across studies	22	Present results of any assessment of risk of bias across studies (see Item 15). [illegible]
Additional analysis	23	Give results of additional analyses, if done (e.g., sensitivity or subgroup analyses [illegible]
**DISCUSSION**		
Summary of evidence	24	Summarize the main findings including the strength of evidence for each main ou [illegible] key groups (e.g., healthcare providers, users, and policy makers).
Limitations	25	Discuss limitations at study and outcome level (e.g., risk of bias), and at review-le [illegible] identified research, reporting bias).
Conclusions	26	Provide a general interpretation of the results in the context of other evidence, ar [illegible]
**FUNDING**		
Funding	27	Describe sources of funding for the systematic review and other support (e.g., su [illegible] systematic review.

From: Moher D, Liberati A, Tetzlaff J, Altman DG, The PRISMA Group (2009). Preferred Reporting Items for Systematic Reviews and Meta-Analyses: The PRISMA Statement. PLoS Med 6(7): e1000097. doi:10.1371/journal.pmed1000097

For more information, visit: **www.prisma-statement.org**.
